# Re Working Hours of Private Nurses

**Published:** 1919-05-31

**Authors:** 


### THE EDITOR'S LETTER-BOX.

[Correspondence on all subjects is invited, but we cannot in any way be responsible for the opinions expressed by our correspondents, who must give their name and address as a guarantee of good faith, but not necessarily for publication. Correspondents are reminded that brevity of style and conciseness of statement greatly facilitate early insertion.]

### Re Working Hours of Private Nurses.

SIR,—In all the good things promised in the general reconstruction of the nursing profession, now so prominently before the public, the nurse working on private cases takes the part of a looker-on, interested, and perhaps hoping, in the general distribution of benefits, a few crumbs may fall to her lot.

The subject brought forward in your "Letter-Box" of shorter hours is a far-reaching but a difficult problem to solve in regard to the private nurse.

The lines of a private nurse, run on a parallel to that of a medical man, who, being a servant of the sick public, is at their service any hour of the day or night, and who finds it difficult to conform to any set rules and control the hours of his working day.

A nurse experienced in private work knows that reasonable relaxation is afforded when the acute stages are past, but to confine her skill and powers within the limitation of an eight-hour day is obviously impossible from every point of view, except where affluence admits a trio. Herein is displayed the essence of vocation predominating the theories and ethics of profession.

Even though the suggestion of a relief nurse was made a workable solution, I doubt if the scheme would prosper. Practical difficulties in many trifling ways would arise, financial, temperamental, domestic, and who would consent to become a relief nurse? Would she receive honour from her *confrères*? Her pathway would be strewn with difficulty, and most certainly she would require to walk circumspectly!

Sympathetic consideration and thought should be expended on behalf of the private nurse in the general rearrangement of conditions, for hers is often an isolated life; yet she forms an integral and substantial portion of the nursing profession. She is the finished article, the turned-out product and representative of every training-school throughout the Empire. From her the thinking public forms its ideas of what a nurse should be. Too often this public takes advantage of her, and misuses her time and her powers. It is for the private nurse that legislation and the one authoritative central control is required, much more so than for those sheltered in institutions. It is hopefully expected that the far-reaching influence of the College will come into direct contact with every member of the great floating body of nurses working in private, especially those attached to no concrete system, *i.e.*, on their own.

There can be no solution for the problem of hours and the betterment of conditions for the nurse in private by rule or by dogma. The solution lies largely in her own hands, and in her individuality, added to by the generosity of the relatives, emphasised in extent, according to the estimate, and the value of the services rendered, aided by the doctor in charge of the case, who should see that the powers of the nurse are not overtaxed and that all reasonable recreation is afforded.

Inculcate into nurses an increased responsibility and deference toward their vocation first, and, secondly, their profession. Educate in the public an increased valuation of the services rendered by them apart from £ s. d., and establish thereby mutual understanding and good will. The solution to the problem thus necessarily lies within the nurse herself, to exert her individual rights to secure to herself necessary recreation and leisure, with due consideration to circumstances, and to secure relief if proportionate leisure is denied to her.

Yours faithfully,

